# Supplementary material for: The RNA secondary structure of androgen receptor-FL and V7 transcripts reveals novel regulatory regions
Source: Nucleic Acids Res. 2024 Mar 30;52(11):6596–613. doi: 10.1093/nar/gkae220 (PMC11194067; doi:10.1093/nar/gkae220)

motif_1.stockholm_1.cacofold.R2R

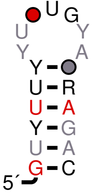

motif_5.stockholm_1.cacofold.R2R

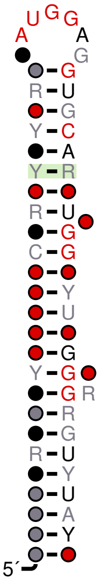

motif_6.stockholm_1.cacofold.R2R

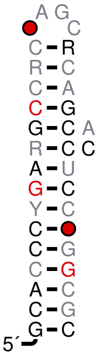

motif_7.stockholm_1.cacofold.R2R

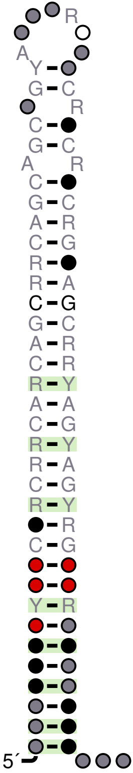

motif_9.stockholm_1.cacofold.R2R

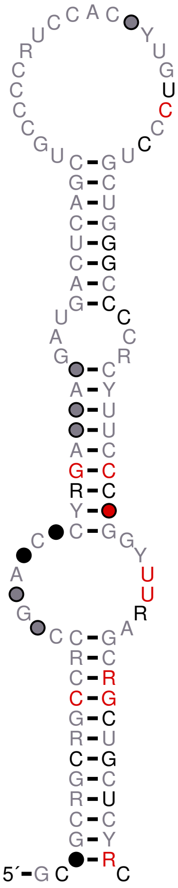

motif_11.stockholm_1.cacofold.R2R

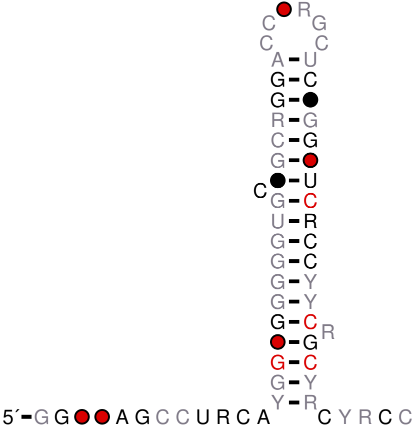

motif_12.stockholm_1.cacofold.R2R

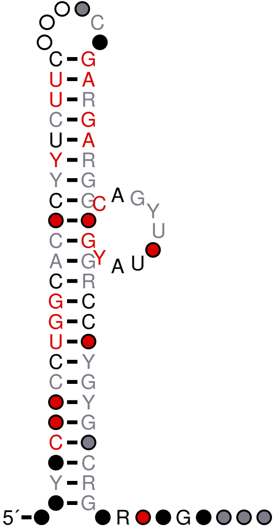

motif_13.stockholm_1.cacofold.R2R

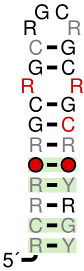

motif_20.stockholm_1.cacofold.R2R

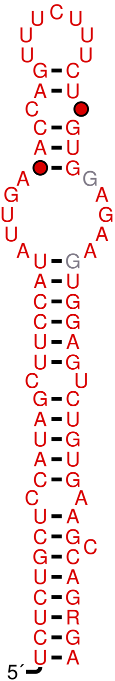

motif_21.stockholm_1.cacofold.R2R

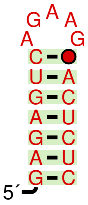

motif_22.stockholm_1.cacofold.R2R

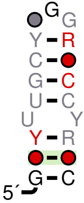

Supplement: gkae220_Supplemental_Files [file gkae220_supplemental_files.zip › Supplementary File S5.zip!/Supplementary File S5/V7_CaCo.zip!/V7_CaCo/All_V7_CaCo.pdf]
